# Arts-based research: Attending to methods

**DOI:** 10.1016/j.metip.2025.100179

**Published:** 2025-06

**Authors:** Cassandra Phoenix, Kerry Chamberlain

**Affiliations:** a Department of Sport and Exercise Sciences, Durham University, Durham, UK; b School of Psychology, Massey University, Auckland, New Zealand

Arts-based research covers a considerable diversity of approaches and uses in research and is not readily amenable to definition, although definitions have been offered. For instance, McNiff (2018, p. 24) has described arts-based research “as a process of inquiry whereby the researcher, alone or with others, engages the making of art as a primary mode of inquiry”. Or as Leavy (2018, p. 4) proposes, arts-based research is “an umbrella category that encompasses all artistic approaches to research”. Some researchers consider arts-based research to be a paradigm (Gerber et al., 2018). This diversity is reflected in the wide range of approaches that arts-based work can encompass, covering visual art forms, (drawing, painting, photography, collage, and other art forms such as film, video, graphic novels, comics, etc.), literary forms (including creative fiction and non-fiction, poetry, scripts, essays, novels, etc.) and performative forms (including theatre, dance, movement, storytelling, etc), as well as other forms that extend beyond these categories (such as quilting, needlework, music, multi-media, and multiple or combined forms). These various forms can be, and have been, put into service in the conduct of research across all phases of research, for problem generation and demarcation, for data collection, for analysis and interpretation, and for dissemination and representation (see Leavy, 2020).

Although arts-based research does not feature that strongly in research publications within psychology, arts-based research still claims significant interest, especially amongst qualitative researchers (Gullion and Schäfer, 2018). For example, an earlier special issue on arts-based research in psychology (Chamberlain et al., 2018) received over 60 submissions, accepting 27 articles for publication in the special issue and a further 14 that were published in subsequent issues of the journal. These articles represented a broad cross-section of artistic approaches and uses, as noted in the following examples. Miller (2018) presented poetry drawn from interview transcripts with people who had recently moved into residential aged care, and Halkovic (2018) wrote a poem to communicate the ethical matters that arose in conducting research where participants revealed traumatic stories. Shanahan (2018) used a zine to document the process of prison visiting. Satchwell and Davidge (2018) co-created a fictional story with a participant in their research to deliver an alternative understanding of autism, and Clarke (2018) presented a multi-voiced story of madness drawn from his personal experiences. Smith (2018) used a photo essay to illustrate the lives of trans, agender, and genderqueer youth. Green (2018) presented portraits of William James, Sigmund Freud, and B. F. Skinner using a word-cloud of words that appeared frequently in one of each man’s influential books. Hansen (2018) used time-lapse video to investigate street art and graffiti as visual dialogue, and Ulmer (2018) presented a photo-essay of street art accompanied by poetic statements to illustrate slow approaches to research and writing, alongside how people communicate through everyday visual interventions. There were many other, equally engaging, articles including 16 pieces of flash fiction depicting aspects of research engagement (Pettit et al., 2018).

There are many reasons offered for employing arts-based methods in research. For example, Coemans and Hannes (2017), reviewing how arts-based methods were used in community-based inquiries with vulnerable populations, found the benefits claimed from the use of arts-based research included increased appeal of the research to participants, engaging participants more readily and more strongly in the research, reducing space and power imbalances between researchers and participants, offering a safe space for the discussion of sensitive issues, creating more useful and interesting data for analysis, with an emphasis on positive rather than negative aspects of experience, capturing material that went beyond the spoken word, and allowing for dissemination in new ways through creating change in policies and audiences. Similarly, Majid and Kandasamy (2021) reviewed arts-based health services research and argued that such research generated projects that were more participatory in nature, and produced knowledge from communities that was effective in informing the design and delivery of interventions.

Other arguments for the use of arts-based research include its value in providing new ways of working that do not rely on accepted Western methods (Seppälä et al., 2021), making it easier to work with indigenous groups (Hammond et al., 2018), marginalised groups (Asakura et al., 2020), hard-to-reach participants (Smyth et al., 2018), and in researching difficult topics, such as incest Pearse-Otene (2021) further, as the research environment changes to value research impact more highly, arts-based research can have considerable value in delivering highly effective forms of dissemination (Boydell et al., 2017), and in delivering impact differently (Parsons et al., 2017). For example, Dunn, O’Keeffe, Stapley, and Midgley (2018) report on how they worked with young people who had experienced depression to coproduce a film about depression and therapy, and how that was widely disseminated on a video-sharing platform.

As with all research, there are debates about value, process, and quality. For instance, Brown (2022) argues that arts-based research does not necessarily produce richer or deeper data and interpretations, but rather different data and interpretations, making it both interesting and valuable to pursue. Greenwood (2019, p. 5) proposes that “Arts-based research needs to be explicit about what is being investigated. If the objective is not clear, then the result may still be art, but it is hard to call it research”, while also noting that this does not imply that research design and methods need to be rigid or static. Although mooted as enhancing participant engagement, it can also be difficult and uncomfortable for participants to engage with arts-based methods, and researchers need to be constantly reflexive and aware of power balances and ethical matters in such research (Seppälä et al., 2021). Phillips et al. (2024) reviewed articles that used arts-based approaches in researching patient and public involvement in healthcare, finding that the time, cost, and effort associated with these methods were limitations to their use, as most methods employed were resource-intensive. They also identified ethical concerns, and a lack of acceptance of this form of investigation as research, as further tensions for their adoption.

Alongside an increasing interest in arts-based research, we can discern a recent rise in publications that discuss the *methods* of artsbased research. For example, Leavy (2020) discusses a range of methodologies, with examples of research practice, Ward and Shortt (2020) discuss the use of arts-based methods for researching businesses and organisations, as well as its use in the humanities, Gerber (2022) discusses the value of imagination and arts-based practices in research, and Hickey-Moody et al. (2021) consider how arts-based methods can be used in research with children. There are also a considerable range of articles discussing specific arts-based research practices, such as Davis’ (2021) arguments for critical poetic inquiry as a culturally relevant methodology, Fleetwood-Smith, Tischler, and Robson’s (2021) commentary on using creative, sensory, and embodied methods in research with people with dementia, or Ottoni, Winters, and Sims-Gould’s (2023) discussion of a digitally mediated photovoice method.

Given *Methods in Psychology* has a specific focus on research methods and methodologies, we considered it timely to develop a special issue where arts-based research methods could receive further attention. The call for the special issue sought articles that promoted the value of artsbased approaches for research in psychology, discussed innovative ways in which arts-based research can be used in psychology, discussed the rationales and challenges in using arts-based research in psychology, critically examined the premises and practices of arts-based methodologies and methods, offered theorised discussions of how arts-based research functions to achieve its goals, or discussed specific aspects of arts-based research, such as improving rigour, enhancing dissemination, and so on.

## Engaging in arts-based research: The special issue

First, we would like to thank the authors and reviewers^1^ of these articles for their time and contributions; without them the special issue could not appear. We were pleased that the special issue attracted a good number of contributions; we received 26 submissions, with a few being ruled out of scope, some others withdrawing after review, and 14 finally accepted for publication^1^. We encouraged submissions from beyond psychology, as we considered that researchers external to the discipline can have useful things to say to psychology researchers. In this we were successful, receiving several submissions from authors located in other disciplines, with interesting and informative argument and process.

This special issue brings together these 14 papers, which span a range of arts-based methods, research areas, disciplines and practice. As individual pieces, and as a collection, these contributions offer rich insight into the rationales, challenges, premises, and practices of arts-based methods and methodologies for psychology.

The issue includes a critical commentary from geographers Boyd and Barry (2024) who illustrate how artistic research differs methodologically and epistemologically from quantitative and qualitative paradigms through its attentiveness to process-orientated questions and knowledges, which often stretch beyond the constrains of language. Several papers foreground the alignment of specific arts-based methods with theoretical approaches underpinning psychology research, in ways that hold potential for extending the field. For example, McGannon et al. (2024) show the value of the hermit crab essay - a relatively under-utilised form of creative non-fiction – to surface experiences of running fathers through narrative inquiry. Similarly, Ritondo et al. (2024) demonstrate how eliciting dialogue using auto-photography (or Photovoice) can be a valuable means of centring participant voices, itself a core principle of feminist theory. Meanwhile, seeking to advance the integration of creative and visual methods within phenomenological psychology research, Day et al. (2024), also using auto-photography methods, report on their analysis in which the visual materials produced by their participants were interpreted separately from the interview data. They argue that such visual displays of analysis (here, in the form of collage) offer new avenues for demonstrating and creating impact from research on living with ‘unseen’ chronic health conditions.

The theme of ‘unseen’ health conditions remains a focal point for several other contributions to this special issue. Sexual and reproductive health (Govender et al., 2024), Covid-19 (Gerber et al., 2024) and mental health (Elliott, 2024; Macdonald et al., 2024 all provide illustrative examples of the possibilities for arts-based research to build collaborative communities (both virtually, and through the necessary cultivation of safe, supportive spaces) and enable culturally specific understandings of experiences where stigma and socio-cultural dynamics can silence discussion.

This, of course, is not to suggest that arts-based research comes without challenges and dilemmas, and we appreciate the learning opportunities provided by many of our contributors for their thoughtful reflections on how and where these can surface. These include Elliott’s (2024) observations regarding the complications of developing the ‘art’ in arts-based research, and Macdonald et al., 2024 work with children who use digital storytelling to share their experiences of anxiety. The articles also extend into broader discussions around both the *ethical value* and *ethical practice* of arts-based approaches, with examples relating to participating in research with vulnerable children (Huard et al., 2024), developing arts-based pedagogy and practice within psychology (Douglas and Carless, 2024), and in its application for supporting those conducting qualitative trauma research (McMahon et al., 2024).

The special issue also foregrounds the perspectives of creative practitioners, with Dumaresq and McFerran (2024) offering an informative synthesis of the literature to help respond to questions surrounding the function and purpose of arts-based research in dance therapy. Creativity is also a focus of the discussion by Margolin et al. (2024) of how arts-based work can unlock the creative wisdom of researchers and participants, and by a weaving of personal stories with professional questions by Frantzich (2024), a psychotherapist exploring ways to extend embodied language and communication through Embodied Theatre Ecology.

Articles in this special issue responded well to the specifics of our call. Overall, the special issue has delivered a valuable contribution to the field, offering some useful, interesting, and at times inspiring approaches to conducting arts-based research. As a group, the articles document the valuable contributions that arts-based methods can deliver in research – how they enhance rapport and connection, minimise power differentials, allow access to different kinds of information, allow for investigation of sensitive topics, and build communities of research. But these articles also make it clear that taking an arts-based approach brings challenges and dilemmas alongside benefits and rewards. We are grateful to the openness and honesty of authors who offered commentary on how these challenges and the complexity of conducting research in this arena was - or indeed could be - responded to. For in research, as we know, what we do determines what we can find out. Perhaps the overarching message is that arts-based methods are diverse and, like any other approach to research, require thoughtful planning and ongoing reflexive engagement in their practice.

These articles, as with any special issue collection, do not offer definitive answers and conclusions to the issues involved in conducting arts-based research. There is still plenty of room for ongoing discussion around the value, contribution and cautions of arts-based methods within psychology. However, we trust that this initial collection published in *Methods in Psychology* will go some way towards encouraging others, both within and beyond psychology, to consider incorporating – either through continued professional development, or collaborating with existing experts in the field - these demanding, yet engaging methods into their future research.

## Footnotes

1 We should note that processing these 26 submissions involved invitations to 112 reviewers, and are grateful to those reviewers who responded in a timely manner, especially those who agreed and provided in-depth reviews, and in many cases re-reviews, of the submissions.
